# Supplementary material for: Metabolic and genomic analysis elucidates strain-level variation in Microbacterium spp. isolated from chromate contaminated sediment
Source: PeerJ. 2015 Nov 10;3:e1395. doi: 10.7717/peerj.1395 (PMC4647564; doi:10.7717/peerj.1395)
Supplement: Supplemental Information 1 [file peerj-03-1395-s003.docx]

**Supporting Information-Tables**

| Table S1. Growth and reduction rates of each strain | | | |  |
| --- | --- | --- | --- | --- |
|  | **Growth Rates^a^** | **Growth Rates with Cr** | **Reduction Rate (μM hr^-1^)** | |
| **Cr-K1W** | 0.7 | 0.1 | 15 | |
| **Cr-K20** | 1.4 | 0.9 | 1 | |
| **Cr-K29** | 1.4 | 1.5 | 37 | |
| **Cr-K32** | 1.5 | 1.6 | 36 | |

^a^Rates are calculated for a 48 hour interval.

| Table S2. General genome information and statistics from IMG annotation | | | | | | | |
| --- | --- | --- | --- | --- | --- | --- | --- |
|  | Genome Status | Coding DNA bases (bp) | DNA scaffolds | GC Content (%) | Protein coding genes | Genes with function prediction | Accession Number |
| Cr-K20 | Draft | 3648231 | 81 | 68.60 | 3804 | 3096 | SAMN02594832 |
| Cr-K32 | Draft | 3585911 | 37 | 68.27 | 3669 | 3009 | SAMN02594834 |
| Cr-K1W | Draft | 3650798 | 36 | 68.61 | 3763 | 3083 | SAMN02594833 |
| Cr-K29 | Draft | 3517029 | 44 | 68.32 | 3616 | 2957 | SAMN02594835 |

| Table S3. Genomic stats from the AbySS assemblies | | | | | | |
| --- | --- | --- | --- | --- | --- | --- |
|  | Genome Status | Genome Size (mBP) | Num. of Contigs | Mean Contig Size (kBP) | Max Contig Size (kBP) | N50 |
| Cr-K1W | Draft | 3.91 | 46 | 85 | 875 | 5 |
| Cr-K20 | Draft | 3.91 | 92 | 42.5 | 488 | 10 |
| Cr-K29 | Draft | 3.79 | 86 | 44 | 485 | 7 |
| Cr-K32 | Draft | 3.86 | 69 | 56 | 570 | 5 |

| Table S4. 35 specific single copy orthologs used for examining genome completeness and the numbers of copies found in each genome. | | | | | |
| --- | --- | --- | --- | --- | --- |
| Function ID | Name | K1W | K20 | K29 | K32 |
| COG0012 | Predicted GTPase, probable translation factor | 1 | 1 | 1 | 1 |
| COG0016 | Phenylalanyl-tRNA synthetase alpha subunit | 1 | 1 | 1 | 1 |
| COG0048 | Ribosomal protein S12 | 1 | 1 | 1 | 1 |
| COG0049 | Ribosomal protein S7 | 1 | 1 | 1 | 1 |
| COG0052 | Ribosomal protein S2 | 1 | 1 | 1 | 1 |
| COG0080 | Ribosomal protein L11 | 1 | 1 | 1 | 1 |
| COG0081 | Ribosomal protein L1 | 1 | 1 | 1 | 1 |
| COG0085 | DNA-directed RNA polymerase, beta subunit | 1 | 1 | 1 | 1 |
| COG0087 | Ribosomal protein L3 | 1 | 1 | 1 | 1 |
| COG0088 | Ribosomal protein L4 | 1 | 1 | 1 | 1 |
| COG0090 | Ribosomal protein L2 | 1 | 1 | 1 | 1 |
| COG0091 | Ribosomal protein L22 | 1 | 1 | 1 | 1 |
| COG0092 | Ribosomal protein S3 | 1 | 1 | 1 | 1 |
| COG0093 | Ribosomal protein L14 | 1 | 1 | 1 | 1 |
| COG0094 | Ribosomal protein L5 | 1 | 1 | 1 | 1 |
| COG0096 | Ribosomal protein S8 | 1 | 1 | 1 | 1 |
| COG0097 | Ribosomal protein L6P/L9E | 1 | 1 | 1 | 1 |
| COG0098 | Ribosomal protein S5 | 1 | 1 | 1 | 1 |
| COG0099 | Ribosomal protein S13 | 1 | 1 | 1 | 1 |
| COG0100 | Ribosomal protein S11 | 1 | 1 | 1 | 1 |
| COG0102 | Ribosomal protein L13 | 1 | 1 | 1 | 1 |
| COG0103 | Ribosomal protein S9 | 1 | 1 | 1 | 1 |
| COG0124 | Histidyl-tRNA synthetase | 1 | 1 | 1 | 1 |
| COG0184 | Ribosomal protein S15P/S13E | 1 | 1 | 1 | 1 |
| COG0185 | Ribosomal protein S19 | 1 | 1 | 1 | 1 |
| COG0186 | Ribosomal protein S17 | 1 | 1 | 1 | 1 |
| COG0197 | Ribosomal protein L16/L10E | 1 | 1 | 1 | 1 |
| COG0200 | Ribosomal protein L15 | 1 | 1 | 1 | 1 |
| COG0201 | Preprotein translocase subunit SecY | 1 | 1 | 1 | 1 |
| COG0256 | Ribosomal protein L18 | 1 | 1 | 1 | 1 |
| COG0495 | Leucyl-tRNA synthetase | 1 | 1 | 1 | 1 |
| COG0522 | Ribosomal protein S4 and related proteins | 1 | 1 | 1 | 1 |
| COG0525 | Valyl-tRNA synthetase | 1 | 1 | 1 | 1 |
| COG0533 | Metal-dependent proteases with possible chaperone activity | 1 | 1 | 1 | 1 |
| COG0541 | Signal recognition particle GTPase | 1 | 1 | 1 | 1 |

| Table S5. Pfam categories from IMG annotations used for the PCA | | | | |
| --- | --- | --- | --- | --- |
|  | Cr-K32 | Cr-K20 | Cr-K1W | Cr-K29 |
| Amino acid transport and metabolism | 133 | 128 | 128 | 127 |
| Carbohydrate transport and metabolism | 127 | 135 | 135 | 121 |
| Cell cycle control, cell division, chromosome partitioning | 14 | 16 | 16 | 12 |
| Cell motility | 20 | 20 | 20 | 19 |
| Cell wall/membrane/envelope biogenesis | 86 | 91 | 91 | 86 |
| Coenzyme transport and metabolism | 80 | 86 | 85 | 80 |
| Defense mechanisms | 12 | 15 | 14 | 11 |
| Energy production and conversion | 101 | 107 | 106 | 99 |
| Function unknown | 177 | 169 | 169 | 176 |
| General function prediction only | 129 | 123 | 123 | 129 |
| Inorganic ion transport and metabolism | 96 | 90 | 90 | 93 |
| Intracellular trafficking, secretion, and vesicular transport | 29 | 27 | 27 | 27 |
| Lipid transport and metabolism | 43 | 49 | 48 | 43 |
| Nucleotide transport and metabolism | 39 | 43 | 43 | 39 |
| Posttranslational modification, protein turnover, chaperones | 45 | 46 | 46 | 45 |
| Replication, recombination and repair | 79 | 82 | 81 | 80 |
| RNA processing and modification | 3 | 3 | 3 | 3 |
| Secondary metabolites biosynthesis, transport and catabolism | 16 | 12 | 12 | 16 |
| Signal transduction mechanisms | 31 | 36 | 36 | 32 |
| Transcription | 111 | 122 | 122 | 110 |
| Translation, ribosomal structure and biogenesis | 125 | 122 | 122 | 125 |
| unclassified | 2130 | 2092 | 2204 | 2092 |

| Table S6. 95 Metabolites used in the Biolog GP2® Plate and the call for 0(-) negative, 1(+) positive, and .05(+/-) borderline for each isolate. | | | | |
| --- | --- | --- | --- | --- |
|  | **Isolate** | | | |
| **Biolog GP2® Metabolite** | **Cr-K20** | **Cr-K29** | **Cr-K32** | **Cr-K1W** |
| α-Cyclodextrin | 0 | 0 | 0 | 0 |
| β-Cyclodextrin | 0 | 0 | 0 | 0 |
| Dextrin | 1 | 1 | 1 | 1 |
| Glycogen | 1 | 1 | 1 | 1 |
| Inulin | 0 | 0 | 0 | 0 |
| Mannan | 1 | 1 | 1 | 1 |
| Tween 40 | 1 | 1 | 1 | 1 |
| Tween 80 | 1 | 1 | 1 | 1 |
| N-Acetyl-D-Glucosamine | 0 | 0 | 0 | 0 |
| N-Acetyl-β-D-Mannosamine | 0.5 | 0 | 0 | 0 |
| Amygdalin | 1 | 1 | 1 | 1 |
| L-Arabinose | 1 | 0 | 0 | 1 |
| D-Arabitol | 0 | 1 | 1 | 1 |
| Arbutin | 0 | 0 | 0 | 0 |
| D-Cellobiose | 1 | 1 | 1 | 1 |
| D-Fructose | 1 | 1 | 1 | 1 |
| L-Fucose | 1 | 0 | 0 | 0 |
| D-Galactose | 1 | 1 | 1 | 1 |
| D-Galacturonic Acid | 0 | 0 | 0 | 0 |
| Gentiobiose | 1 | 1 | 1 | 1 |
| D-Gluconic Acid | 0.5 | 0 | 0 | 1 |
| α-D-Glucose | 1 | 1 | 1 | 1 |
| m-Inositol | 0.5 | 0 | 0 | 0 |
| α-D-Lactose | 0 | 0 | 0 | 0 |
| Lactulose | 0 | 0 | 0 | 0 |
| Maltose | 1 | 1 | 1 | 1 |
| Maltotriose | 1 | 1 | 1 | 1 |
| D-Mannitol | 1 | 1 | 1 | 1 |
| D-Mannose | 1 | 1 | 1 | 1 |
| D-Melezitose | 0 | 0 | 0.5 | 0 |
| D-Melibiose | 0 | 0 | 0 | 1 |
| α-Methyl-D-Galactoside | 0 | 0 | 0 | 0.5 |
| β-Methyl-D-Galactoside | 0 | 0 | 0 | 0 |
| 3-Methyl D-Glucose | 1 | 1 | 1 | 1 |
| α-Methyl-D-Glucoside | 1 | 1 | 1 | 0.5 |
| β-Methyl-D-Glucoside | 0.5 | 0 | 0 | 1 |
| α-Methyl-D-Mannoside | 0.5 | 0 | 0.5 | 1 |
| Palatinose | 1 | 1 | 1 | 1 |
| D-Psicose | 0.5 | 0 | 0 | 1 |
| D-Raffinose | 0.5 | 0 | 0 | 0 |
| L-Rhamnose | 1 | 0 | 0 | 0 |
| D-Ribose | 1 | 1 | 1 | 1 |
| Salicin | 1 | 1 | 1 | 1 |
| Sedoheptulosan | 0 | 0 | 0 | 0 |
| D-Sorbitol | 1 | 1 | 1 | 1 |
| Stachyose | 0 | 0 | 0 | 0 |
| Sucrose | 1 | 1 | 1 | 1 |
| D-Tagatose | 0.5 | 0.5 | 0.5 | 0.5 |
| D-Trehalose | 1 | 1 | 1 | 1 |
| Turanose | 1 | 1 | 1 | 1 |
| Xylitol | 1 | 0 | 0.5 | 1 |
| D-Xylose | 1 | 0 | 0 | 1 |
| Acetic Acid | 1 | 0 | 0.5 | 0 |
| α-Hydroxybutyric Acid | 1 | 1 | 1 | 0 |
| β-Hydroxybutyric Acid | 0 | 0 | 0 | 0 |
| γ-Hydroxybutyric Acid | 0 | 0 | 0 | 0 |
| p-HydroxyPhenylacetic Acid | 1 | 1 | 1 | 0 |
| α-Ketoglutaric Acid | 0 | 0 | 0 | 0 |
| α-Ketovaleric Acid | 1 | 1 | 1 | 0 |
| Lactamide | 0 | 0 | 0 | 0 |
| D-Lactic Acid Methyl Ester | 1 | 1 | 1 | 0 |
| L-Lactic Acid | 1 | 0 | 1 | 0 |
| D-Malic Acid | 0 | 0 | 0 | 0 |
| L-Malic Acid | 0 | 0 | 0 | 0 |
| Pyruvatic Acid Methyl Ester | 1 | 1 | 1 | 1 |
| Succinic Acid Mono-methyl Ester | 1 | 0 | 1 | 0 |
| Propionic Acid | 1 | 1 | 1 | 0 |
| Pyruvic Acid | 1 | 1 | 1 | 1 |
| Succinamic Acid | 0 | 0 | 0 | 0 |
| Succinic Acid | 0.5 | 0 | 0 | 0 |
| N-Acetyl-L Glutami cAcid | 1 | 0 | 0 | 0 |
| L-Alaninamide | 1 | 1 | 1 | 0 |
| D-Alanine | 0 | 0 | 0 | 0 |
| L-Alanine | 1 | 1 | 1 | 0 |
| L-AlanylGlycine | 1 | 1 | 1 | 0 |
| L-Asparagine | 1 | 1 | 1 | 0 |
| L-Glutamic Acid | 1 | 1 | 1 | 0 |
| Glycyl-L Glutamic Acid | 1 | 0 | 0 | 0 |
| L-Pyroglutamic Acid | 1 | 1 | 1 | 0 |
| L-Serine | 1 | 1 | 1 | 0 |
| Putrescine | 1 | 1 | 1 | 0 |
| 2,3-Butanediol | 0.5 | 0 | 0 | 0 |
| Glycerol | 1 | 1 | 1 | 1 |
| Adenosine | 1 | 1 | 1 | 1 |
| 2'-Deoxy Adenosine | 1 | 1 | 1 | 1 |
| Inosine | 1 | 1 | 1 | 1 |
| Thymidine | 1 | 1 | 1 | 1 |
| Uridine | 1 | 1 | 1 | 1 |
| Adenosine-5'-Monophosphate | 1 | 1 | 1 | 1 |
| Thymidine-5'-Monophosphate | 1 | 1 | 1 | 1 |
| Uridine-5'-Monophosphate | 1 | 0 | 0 | 1 |
| D-Fructose-6-Phosphate | 1 | 0 | 0 | 0 |
| α-D-Glucose-1-Phosphate | 0.5 | 0 | 0 | 0 |
| D-Glucose-6-Phosphate | 1 | 0 | 0 | 1 |
| D-L-α-Glycerol | 1 | 1 | 1 | 1 |
